# Challenges in the Microbiological Diagnosis of Implant-Associated Infections: A Summary of the Current Knowledge

**DOI:** 10.3389/fmicb.2021.750460

**Published:** 2021-10-29

**Authors:** Alessandra Oliva, Maria Claudia Miele, Dania Al Ismail, Federica Di Timoteo, Massimiliano De Angelis, Luigi Rosa, Antimo Cutone, Mario Venditti, Maria Teresa Mascellino, Piera Valenti, Claudio Maria Mastroianni

**Affiliations:** ^1^Department of Public Health and Infectious Diseases, Sapienza University of Rome, Rome, Italy; ^2^Department of Biosciences and Territory, University of Molise, Pesche, Italy

**Keywords:** implant-associated infection, biofilm, sonication, culture-based methods, diagnosis, BioTimer Assay, molecular methods, metabolic assays

## Abstract

Implant-associated infections are characterized by microbial biofilm formation on implant surface, which renders the microbiological diagnosis challenging and requires, in the majority of cases, a complete device removal along with a prolonged antimicrobial therapy. Traditional cultures have shown unsatisfactory sensitivity and a significant advance in the field has been represented by both the application of the sonication technique for the detachment of live bacteria from biofilm and the implementation of metabolic and molecular assays. However, despite the recent progresses in the microbiological diagnosis have considerably reduced the rate of culture-negative infections, still their reported incidence is not negligible. Overall, several culture- and non-culture based methods have been developed for diagnosis optimization, which mostly relies on pre-operative and intra-operative (i.e., removed implants and surrounding tissues) samples. This review outlines the principal culture- and non-culture based methods for the diagnosis of the causative agents of implant-associated infections and gives an overview on their application in the clinical practice. Furthermore, advantages and disadvantages of each method are described.

## Introduction

Implant-associated infections (IAIs) are associated with high morbidity and increased costs for the healthcare systems (Hedrick et al., 2006). IAIs, including, amongst other, those associated with totally intracorporeal devices [Prosthetic Joint Infections (PJIs), Cardiovascular Implantable Electronic Device Infections (CIED-Is), Neurosurgical Infections (NS-Is), Ureteral Stent Infections (US-Is), Vascular Graft Infections (VG-Is), and Breast Implant Infections (BI-Is)], are characterized by microbial biofilm formation on implant surface, which makes the microbiological diagnosis difficult and requires a complete device removal for their correct management (Deng and Lv, 2016).

In general, implants are made of synthetic abiotic material or devitalized biological structures. Furthermore, medical devices are either crossing the anatomic barriers (i.e., central venous or urologic catheters, dental implants) or are totally intracorporeal (i.e., orthopedic, neurosurgical, cardiac, vascular implants), with the latter group being classified as intravascular and extravascular implants. The pathogenesis of these various devices as well as the interaction with the host are quite different. Indeed, while intravascular implants mainly interact with coagulation factors and circulating blood cells, extravascular implants interact with surrounding tissue, interstitial fluid, and attracted phagocytes, in the absence of direct interaction with the circulating blood (Zimmerli and Sendi, 2011).

Although many progresses have been made, the diagnosis of the causative microorganisms of IAIs still remains challenging. In fact, diagnostic sensitivity might be reduced since not all the techniques are able to completely detach biofilm from removed implants and the antibiogram is generally performed on planktonic form of detached bacteria (Drago, 2017). Furthermore, a previous antimicrobial therapy might influence the diagnostic yield of culture-based methods (Xu et al., 2017).

In the last years, several approaches have been developed to overcome the above-mentioned limitations, including sonication of the implants before culture, molecular assays, methods based on bacterial metabolism in the biofilm or their combinations (Høiby et al., 2015).

In this report, the application of culture- and non-culture based methods for the diagnosis of causative agents of IAIs will be reviewed, with a main focus on infections of totally intracorporeal devices. In fact, although also catheter-related infections are characterized by biofilm formation, the diagnostic methods between infections associated to devices crossing anatomic barriers and those totally implanted are quite different.

## Literature Search

The online database PubMed was searched using the following terms: “implant-associated-infections,” “biofilm-infections,” “microbiological diagnosis,” AND “implant-associated-infections” OR “biofilm-infections,” “sonication,” “molecular analyses,” AND “implant-associated-infections” OR “biofilm-infections,” “metabolic assays,” AND “implant-associated-infections” OR “biofilm-infections,” “tissue culture,” AND “implant-associated-infections” OR “biofilm-infections,” “Resazurin Assay,” “BioTimer Assay,” “XTT Assay,” “Gram stain,” “microscopy,” “Broad-range 16S rRNA gene PCR” AND “implant-associated-infections” OR “biofilm-infections,” “sequencing” AND “implant-associated-infections” OR “biofilm-infections,” “Multiplex PCR” AND “implant-associated-infections” OR “biofilm-infections,” “IBIS T5000” AND “implant-associated-infections” OR “biofilm-infections,” “dithiothreitol.” The combination of the abovementioned terms with “Cardiac Device Infections,” “CIED infections,” “Ureteral Stent infections,” “Prosthetic Joint Infections,” “Neurosurgical Infections,” “Vascular Graft Infections” and “Breast Implant Infections” was also used.

## Culture-Based Methods

### Swabs and Tissue Cultures

Tissue swabs are never indicated for the diagnosis of IAIs due to their lower sensitivity compared to tissue culture (Dy Chua et al., 2005; Drago et al., 2019). Furthermore, superficial swabs (i.e., from fistula) can be easily contaminated by normal skin flora, therefore not representing the true pathogen and possibly contributing to an erroneous etiological diagnosis.

On the other hand, cultures from the tissue (TC, tissue culture) adjacent to the device are part of the diagnostic approach toward IAIs (Peel et al., 2017).

### Sonication

#### Method

**FIGURE 1:**
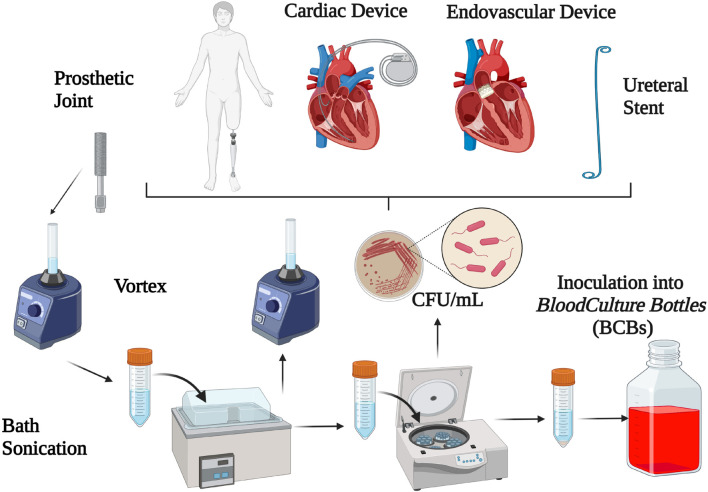
Overview of sonication method for the diagnosis of implant-associated infections.

Sonication is a quantitative method based on the application of long-wave ultrasounds (frequencies above the range of human hearing, 20 kHz) which has been increasingly used in order to enhance bacterial growth by liberating sessile organisms embedded in biofilm (Nguyen et al., 2002; Klug et al., 2003; Carmen et al., 2005; Bjerkan et al., 2009; Rieger et al., 2009; Sampedro et al., 2010; Bonkat et al., 2011). Technically, ultrasound waves radiate through a liquid media and produce high- and low-pressure areas. During the low-pressure phase, microscopic bubbles form and further collapse during the high-pressure phase by releasing a high amount of energy on the surface of the foreign body, which is able to dislodge bacteria from the device (Pitt and Ross, 2003; Trampuz et al., 2003). Sonication is also able to lyse bacterial cells, and whether bacteria are dislodged from foreign bodies or lysed depends on several factors such as acoustic frequency, energy, temperature and time of ultrasound exposure, duration of sonication and the shape of bacteria (Osmon et al., 2013). Among different sonication protocols (Tande and Patel, 2014), the most widely used for dislodging bacteria from foreign bodies are based on 1-min (Trampuz et al., 2007; Hoekstra et al., 2020) or 5-min duration of sonication at power of 0.22 ± 0.04 W/cm^2^ (McDowell and Patrick, 2005; Sampedro et al., 2010; Oliva et al., 2013, 2016), with or without the centrifugation as a concentration process. The process of sonication is depicted in Figure 1.

As a matter of fact, the use of sonication in the clinical microbiology laboratory has been investigated for the diagnosis of PJIs, CIED-Is, NS-Is, VG-Is, and US-Is and is therefore reviewed in the following paragraphs.

#### Sonication for Prosthetic Joint Infection

PJIs represent an important complication of prosthetic surgery, occurring in 1–2% after primary hip or shoulder arthroplasty, 2–4% after knee arthroplasty and up to 9% after elbow arthroplasty (Izakovicova et al., 2019). Microbiological diagnosis of PJIs mostly relies on pre-operative (i.e., synovial fluid cultures) and intra-operative (i.e., tissue culture and implant sonication) samples (Osmon et al., 2013). The sensitivity of synovial fluid culture ranges from 45 to 75%, with a specificity of 95% (Izakovicova et al., 2019); however, up to one-third of intra-operatively culture-positive episodes were negative in pre-operative synovial fluid culture (Schulz et al., 2021).

Sonication was popularized as a diagnostic tool for PJIs by Trampuz et al. (2007), where authors were able to (i) demonstrate higher sensitivity of sonication than TC, (ii) find an optimal cut-off differentiating infection from non-infection, and (iii) demonstrate that the sensitivity of sonication was not hampered by a previous antibiotic therapy (Trampuz et al., 2007). Afterward, several studies have investigated the diagnostic performance of sonication in this setting and, according to the International Consensus Meeting on PJIs in 2018, sonication was recommended as an important element in PJIs diagnostics (Parvizi et al., 2018).

So far, two meta-analyses investigating the sensitivity and specificity of sonication (Zhai et al., 2014; Liu et al., 2017) have been performed. The first was conducted by Zhai et al. (2014) including 12 studies and showing a pooled sensitivity and specificity of 80 and 95%, respectively. Subgroup analyses showed that (i) a 14-day anaerobic culture may improve sensitivity, (ii) the use of centrifugation or vortexing may improve specificity, (iii) using high amount or Ringer’s solution for containers may improve sensitivity and specificity, and (iv) the best sonication fluid culture (SFC) cut-off was > 5 Colony-Forming Units (CFUs) (Zhai et al., 2014). These results were further confirmed by Liu et al. including 16 studies with a pooled sensitivity and specificity of sonication for the diagnosis of PJI of 79 and 95%, respectively. High variability in the method amongst included studies was, however, detected, i.e., for different definitions of PJIs, sample size (59–434 subjects), application of vortexing or centrifugation (11 out of 16 studies), cut-off (not applicable in 6 studies, ranging from 1 to 100 CFU in the other studies) and especially duration of incubation (5–30 days) (Liu et al., 2017). Furthermore, among these studies, 6 compared SFC with TC and overall showed higher sensitivity (but not specificity) of SFC than that of TC and, for patients receiving antimicrobials, this better diagnostic performance was confirmed. Subgroup analyses showed that the specificity of the method may be improved by applying vortexing and centrifugation steps.

Nevertheless, while most studies are in accordance with the superiority of sonication over TC (Portillo et al., 2014; Rothenberg et al., 2017; Renz et al., 2018), several studies have also found the contrary (Van Diek et al., 2017; Grosso et al., 2018). As a matter of fact, Dudareva et al. (2018) demonstrated that the sensitivity of TC was higher than sonication (69% vs. 57%). These findings were further confirmed by a recent study showing higher sensitivity of TC than sonication (94.3% vs. 80.5%), although a certain diagnosis of PJIs was only possible throughout SFC in a not-negligible rate of cases (9%) (Hoekstra et al., 2020). Even more recently, Rieber et al. (2021) showed that the overall sensitivity of TC and SFC was similar (91.3% vs. 90.8%, respectively) and, surprisingly, TC showed significantly better results than SFC in detecting polymicrobial infections (97.0% vs. 67.0%).

In the never-ending debate on whether SFC is better than TC or *viceversa*, it should be noticed that the divergence in results between studies could be attributed to the variability of study conditions, rendering the interpretation of all data even more challenging (Trampuz et al., 2007; Piper et al., 2009; Bjerkan et al., 2012; Borens et al., 2013; Dudareva et al., 2018; Sandbakken et al., 2020). Furthermore, the diagnostic performance of the method also depends on the adopted criteria for defining infection; for instance, Bellova et al. (2019) found different sensitivity and specificity of SFC and TC when using the European Bone and Joint Infection Society (EBJIS) or the International Consensus Meeting (ICM) 2018 definitions, respectively (Bellova et al., 2019).

In light of this, since both sensitivity and specificity of sonication for the diagnosis of PJIs may be influenced by some parameters such as incubation time, previous antibiotic therapy, type of infection (acute or chronic) and the CFU cut-off defined, studies investigating these parameters are following reviewed.

##### Duration of Incubation

Duration of incubation is an important parameter influencing the diagnostic performance of sonication; in fact, on one side a too short incubation may lower bacterial detection, especially when considering low-virulent ones, on the other side a too long incubation may promote contamination of the medium and, therefore, alter the results (Esteban et al., 2013).

However, there has been debate about the optimal length of incubation of PJI samples and systematic assessment of culture duration has not been defined yet (Virolainen et al., 2002; Neut et al., 2003; Williams et al., 2004; Nelson et al., 2005; Parvizi et al., 2006; Trampuz and Widmer, 2006; Trampuz et al., 2007; Piper et al., 2009; Holinka et al., 2011; Esteban et al., 2013; Portillo et al., 2013, 2014; Shen et al., 2015; Renz et al., 2018). Several authors have recommended incubation for 10–14 days in order to improve the sensitivity (Butler-Wu et al., 2011; Minassian et al., 2014; Portillo et al., 2015; Hoekstra et al., 2020; Rieber et al., 2021) whereas only one study, to our knowledge, proposed 30 days of incubation to detect anaerobic bacteria (Esteban et al., 2008). A 2-week incubation period seems to be optimal since early detected species (mostly Staphylococci) emerge predominantly during the first week, whereas late-detected agents (mostly *Cutibacterium* species, formerly known as *Propionibacterium* spp.) are detected mainly during the second week of incubation (Lutz et al., 2005; Schäfer et al., 2008; Portillo et al., 2014). Interestingly, microorganisms grow faster in SFC than in TC. With this regard, Portillo et al. (2014) showed that a difference in bacterial detection between SFC and TC already emerged after 2 days of incubation (48% vs. 26%) and this difference was even more evident after one and 2 weeks of incubation (77% vs. 59% and 81% vs. 61%), respectively.

Recently, Talsma et al. (2021) found that, despite a similar median time to pathogen detection between acute and chronic PJIs (2 days), in acute PJIs all isolates grew within 5 days and therefore a prolonged incubation time may not be necessary. In contrast, for chronic PJIs the time for bacterial growth was longer (11 days) and therefore prolonged incubation appears crucial. When comparing SFC with TC depending on the time of infection (acute vs. chronic PJIs), the same authors found that in acute infections the time to bacterial growth was similar between the 2 methods whereas SFC exhibited a faster pathogen detection than TC in chronic infections (78% vs. 52% after 2 days) (Talsma et al., 2021). Should these results be confirmed, there is the potential to reduce the workload of handling PJI cultures in the laboratory and, consequently, the diagnostic costs according to the time of infection.

##### Effect of Antimicrobial Therapy

One of the major challenges in the diagnosis of PJIs is the possibility that a previous antimicrobial therapy may hamper the diagnostic sensitivity of the method. With this regard, several studies investigated the sensitivity of SFC compared with that of TC in patients receiving antimicrobial therapy up to 14 days before specimen collection and authors found that, despite the sensitivity of SFC is overall reduced in subjects receiving antimicrobial therapy, still SFC was more sensitive (Trampuz et al., 2007; Holinka et al., 2011; Portillo et al., 2013, 2014, 2015; Liu et al., 2017). As a matter of fact, in the review from Liu et al. (2017) in patients who received antibiotic therapy within 14 days sonication performed better than traditional TC. Yan et al. (2018) showed a similar sensitivity of SFC in patients who had received antibiotics and those who had not within 4 weeks before surgery (76.3% vs. 71.2%) and in the recent study from Schulz et al. (2021) the administration of antibiotics did not show any effect on the diagnostic microbiological yield. In detail, when microbiological and non-microbiological diagnostic tests were considered together, the positivity rate was 98 and 97% with and without antibiotics, whereas when considering only SFC the pathogen detection rate was 82% in presence of antibiotics compared to 74% of TC (Schulz et al., 2021).

When taking into account the colony count cut-off, Portillo et al. (2013) found that sensitivity of sonication fluid cultures was significantly lower if patients had previously received antibiotics. As a matter of fact, using the cut-off of 50 CFU/mL, SFC showed a high discriminative power for differentiating between infection and non-infection (Izakovicova et al., 2019), whereas in patients who had received antimicrobials previous to surgery this cut-off should not be used and, consequently, any growth in SFC from patients who had taken antibiotics within 2 weeks from sample collection should be considered positive (Portillo et al., 2013; Stylianakis et al., 2018). Another interesting aspect was that a previous antimicrobial treatment reduced the culture sensitivity of sonication fluid more in acute than in chronic PJIs (Portillo et al., 2013). This finding may be easily explained by the fact that antimicrobial therapy mostly acts on planktonic bacteria, which are more present in acute infections, whereas the killing efficacy of antimicrobials is reduced in chronic infections, which are characterized by a more biofilm formation and, consequently, the diagnostic performance of SFC is augmented (Portillo et al., 2014).

##### Sonication Fluid Inoculated Into Blood Culture Bottles

A promising approach to increase sensitivity is represented by the inoculation of SFC into BCBs. As a matter of fact, a recent meta-analysis including 4 studies (Janz et al., 2013; Portillo et al., 2015; Shen et al., 2015; Stylianakis et al., 2018) showed a pooled sensitivity and specificity of 0.85 and 0.86, respectively (Li et al., 2018), even in patients receiving antibiotics (Portillo et al., 2015). This was especially true considering that growth media in blood culture bottles contain antimicrobial removal systems and therefore allows growth of microorganisms immediately after inoculation.

Inoculating SFC into BCBs was also able to reduce the time of microorganism detection (2.9 vs. 4.2 days) (Janz et al., 2017). Likewise, Portillo et al. (2015) showed that the incubation time was shorter with SFC-BCB than periprosthetic TC and conventional sonication method (72% vs. 18% and 28% after 1 day of incubation, respectively).

However, one of the main limitations of this method is represented by the absence of defining a colony count threshold to define positive culture, therefore influencing the specificity of the method (Jan et al., 2013; Portillo et al., 2015; Stylianakis et al., 2018). Shen et al. (2015) compared SFC in BACTEC bottles with synovial fluid cultures in BACTEC bottles and showed that (i) SFC-BCBs detected a higher number of pathogens than synovial fluid-BCBs, (ii) the sensitivity of SFC-BCBs was higher than that of synovial fluid cultures-BCBs (88% vs. 64%) independently of receiving antimicrobial therapy, and (iii) the specificity of SFC-BCBs was lower than that of synovial fluid cultures-BCBs (87% vs. 98%). Likewise, Janz et al. (2017) found a lower percentage of positive cultures in synovial fluid-BCBs than SFC-BCBs (22% vs. 44%), but the average duration of positive growth in synovial fluid was shortened to 1.8 days, compared with 2.9 days in SF (Janz et al., 2017).

Nevertheless, Rieber et al. (2021) showed that SFC-BCBs is less efficient if anaerobes are the suspected cause of infection and therefore recommended caution when dealing with anaerobes possibly causing PJIs until a gold standard for laboratory handling of anaerobes has been established. Furthermore, the same authors showed that using inoculation into thioglycollate broth was better than into BCBs (Rieber et al., 2021).

#### Sonication for Implant-Associated Infections Other Than Prosthetic Joint Infections

##### Cardiac Implantable Electronic Device Infection

CIED-Is are dangerous conditions with an increasingly incidence over the last years and a significant rate of mortality.

Sonication has been investigated as a diagnostic tool for the diagnosis of CIED-Is, showing overall higher sensitivity than traditional cultures (Rohacek et al., 2010, 2015; Mason et al., 2011; Oliva et al., 2013, 2018; Inacio et al., 2015; Nagpal et al., 2015; Tascini et al., 2016) and, according to the recent guidelines (Blomström-Lundqvist et al., 2020) is considered as an useful diagnostic tool for the etiological diagnosis of CIED-Is, although no definite evidence is provided (Viola et al., 2009). A major study conducted by our group showed that in a total of 20 subjects with clinically defined CIED-Is, SFC was positive in 18/20 (90%) patients in contrast to conventional culture and surgical swab (16/20, 80% and 6/20, 30%, respectively) (Oliva et al., 2013), thus confirming the results obtained by Mason et al. (2011). Subsequently, Nagpal et al. (2015) and Rohacek et al. (2015) demonstrated that bacterial growth was more frequent after sonication than with traditional cultures including swabs, TCs and BCs (Nagpal et al., 2015; Rohacek et al., 2015). Interestingly, sonication was the only method that detected bacteria in four patients (Rohacek et al., 2015) and showed a higher pathogen detection rate in patients on antibiotic therapy than TC (Inacio et al., 2015; Oliva et al., 2018).

In addition, sonication can provide information not only on the detection of the causative pathogen of CIED-Is, but also on its pathogenesis, by evaluating the different rate of pathogen detection according to the different samples analyzed (i.e., generators vs. electrodes) (Oliva et al., 2013, 2018; Rosa et al., 2019).

The diagnostic accuracy of SFC in comparison with 16S rRNA PCR/sequencing on sonication fluid for infected (*n* = 278) and non-infected (*n* = 44) CIEDs has been recently investigated by Esquer Garrigos et al. (2020). Authors found that the sensitivity of 16S rRNA PCR/sequencing was higher than SFC (64% vs. 57.5%, confirmed when considering only definite infections, 76.4% vs. 69.3%), with a similar high specificity (97.7% vs. 95.4%). Interestingly, 16S rRNA PCR/sequencing detected a potential pathogen in a not-negligible rate of culture-negative samples (23.7%).

##### Sonication for Infections After Neurosurgery

Microbiological diagnosis of infections after neurosurgery is essential and it is mainly based on the cerebrospinal fluid (CSF) culture, which, however, can be negative in 23–78% of patients, especially those receiving antibiotics (Martin et al., 2018), combined with the analysis of the implants, when removed. Following the favorable experience with sonication in the setting of other IAIs, different studies evaluated the diagnostic performance of this method in the setting of external ventricular drains (EVDs) and ventriculo-peritoneal (VP) shunts infections (Jost et al., 2014; Prinz et al., 2019; Apostolakis, 2020; Conen et al., 2020). Jost et al. (2014) compared sonication with CSF cultures in 27 explanted devices (14 EVDs, 13 VPSs). In the EVD group, culture after sonication grew significantly more bacteria than the aspirated ventricular CSF cultures (64% vs. 14%), whereas in the VPS group the difference was not significant. Interestingly, the development of clinical significant meningitis might be anticipated by the positivity of EVD or VPS sonication culture.

A not recent study investigating the rate of bacterial colonization in cerebral catheters by using roll-plate or sonication method found that both antibiotic impregnated and non-impregnated catheters were colonized whereas CSF cultures were positive only in a minority of patients (Zabramski et al., 2003). In the study authored by Prinz et al. (2019), tissue homogenate, CSF, and deep swabs were collected for microbiological examination and, in a subset of patients, the removed implants were also sonicated (*n* = 22). Sonication cultures showed a positive microbiological result in the totality of cases (100%), while with the combination of conventional microbiological methods the responsible organism was identified in 60% of the samples (tissue homogenate 57.7%, deep swabs 71.4%, CSF 19.4% each, respectively). Interestingly, in those patients receiving antimicrobial treatment before device explantation, SFC showed 100% of sensitivity compared to 50% of conventional methods, suggesting its use in the clinical practice of EVD and VP infections. The difference between sonication and conventional methods was more evident in the case of low-virulent pathogens (sensitivity 100% vs. 55% in sonication and conventional cultures, respectively).

In the study authored by Roethlisberger et al. (2018) bacterial growth was observed in 19 ventricular EVDs and 21 subcutaneous EVDs throughout sonication of the subcutaneous portion of the catheter and of its tip, the main pathogens being CoNS and *C. acnes.*

Apostolakis (2020) performed a meta-analysis including 6 studies (4 involving EVDs or VP, 1 cranioplasty, 1 spinal fusion instrumentation) with the aim to assess the efficiency of sonication in the diagnostic work-up of postoperative infections following NS. Potential superiority of sonication over conventional microbiologic methods was found in the detection of gram-positive bacteria and in particular of CoNS, with an overall sensitivity of 0.87 and a specificity of 0.57 (Apostolakis, 2020).

##### Sonication for Vascular Graft Infections

VG-Is, although rare, are associated with high morbidity and mortality and the success of antibiotic treatments relies on early and accurate diagnosis (Lyons et al., 2016). However, conventional reference microbiological methods have a low sensitivity rate, as up to 45% of VG-Is still remain culture negative (Legout et al., 2012). Tollefson et al. (1987) first evaluated biofilm breakdown by sonication in an animal model of contamination and in 7 graft materials excised from patients undergoing femoral anastomotic pseudoaneurysm repair. Sonication significantly increased the incidence of positive cultures of graft material compared with broth and blood agar plate culture techniques (Tollefson et al., 1987). In the following years, only few studies investigated the potential diagnostic role of sonication in the setting of VGIs, in combination with molecular methods (Puges et al., 2018; Ulcar et al., 2018). A retrospective study in 2017 highlighted the importance of SFC in parallel with broad-PCR, as they contribute to the optimization of antimicrobial treatment. Indeed, in a total of 22 patients with VG-Is, preoperative BCs were positive in 35.3%, intraoperative TCs in 31.8%, SFC in 79.2%, and PCR from sonicated fluid in 66.7% (Ulcar et al., 2018). Similarly, Puges et al. (2018) compared conventional bacterial cultures with and without prior sonication of specimens and a genus-specific PCR analysis targeted to the most frequent bacteria involved in VG-Is. The sensitivity of the graft culture was 85.7%, of the SFC was 89.7%, and of the genus-specific PCR was 79.5%, respectively. The combination of SFC and PCR achieved a microbiological diagnosis for all patients with VGIs, with a sensitivity of 100% and a specificity of 83.3% (Puges et al., 2018).

##### Sonication for Ureteral Stent Infections

Ureteral stents represent a significant inherent risk of microbial colonization and biofilm formation because they provide an ideal surface for microbial adherence (Joshi et al., 2003; Bonkat et al., 2011; Scotland et al., 2019). Diagnosis of microbial colonization of the ureteral stent (MUSC) is difficult because the cultural methods normally used are not useful in detecting microorganisms embedded in biofilm and consequently a negative urine culture does not rule out biofilm formation. Bonkat et al. (2011) developed a sonication system based on the method described by Trampuz et al. (2007) and showed that SFC detected MUSC in 36% of 408 stents and 93 were positive with sonication alone compared to 8 positive with urine culture alone (Bonkat et al., 2011). The importance of sonication in MUSCs was confirmed by a second study performed by the same group in which the yield of microbial growth using sonication was significantly higher than that observed in urine cultures (Bonkat et al., 2012).

Subsequently, the same research group performed a prospective randomized study comparing the roll-plate technique with sonication in the diagnosis of MUSC. The roll-plate technique showed a higher detection rate of MUSC than sonication and CUC (35% vs. 28 and 8%, respectively). This study demonstrated the superiority of the roll-plate technique, but still confirmed the efficiency of sonication in identifying mixed biofilms (Bonkat et al., 2013).

##### Sonication for Breast Implant Infections

Breast implants are widely used for cosmetic purposes and after mastectomy. Apart from clinical evident infection, a common complication after breast surgery with prosthesis implantation is capsular contracture (Spear and Baker, 1995), whose etiology remains still unclear, although bacterial colonization and biofilm formation by CoNS, *C. acnes*, and other skin-flora microorganisms with consequent low-grade infection are considered the causative mechanism (Pajkos et al., 2003; Del Pozo et al., 2009). Indeed, a statistically significant correlation between a positive culture and symptomatic capsular contracture was found in several studies (Dobke et al., 1995; Ahn et al., 1996; Virden et al., 2020), especially when using sonication as a diagnostic method (Pajkos et al., 2003; Del Pozo et al., 2009; Rieger et al., 2009, 2013, 2016; Reischies et al., 2017). Pajkos et al. (2003) performed a study including implants and capsules removed from patients with or without capsular contracture and showed that the majority of samples obtained from patients with contracture yielded bacteria, significantly higher than in samples obtained from patients without contracture. Del Pozo et al. (2009) analyzed 45 breast implants removed for reasons other than overt infection including capsular contracture (27/45, 60%) and demonstrated that there was a significant association between capsular contracture and the presence of bacteria on the implant. In the following years, Rieger et al. (2013) published a multicentric study including 121 removed implants and, again, a strong correlation between the degree of capsular contracture and positive sonication culture was shown. Subsequently, Karau et al. (2013) prospectively included 328 breast tissue expanders removed for any reason including infection and, apart from showing that in the infection group (*n* = 7) sonication showed higher sensitivity than tissue cultures, demonstrated that a not-negligible rate of breast tissue expanders (16%) appeared to be asymptomatically colonized with normal skin flora. More recently, Reischies et al. (2017) showed a high sensitivity of sonication of implants removed for reasons other than infection and, more interestingly, noticed that the microorganisms isolated (CoNs, *C. acnes*) and suspected to trigger the formation of capsular contracture were not adequately targeted by the common antibiotics used for prophylaxis.

#### Conclusive Remarks of Sonication Method in the Diagnosis of Implant-Associated Infections

Overall, the use of SFC, alone of combined with molecular or metabolic methods or with inoculation into BCBs, exhibited a high performance for the etiological diagnosis of IAIs. Although the majority of data come from PJIs, increasing interest has been shown also for other types of IAIs including endovascular, neurosurgical and ureteral stent ones. The main reason for the high sensitivity of this method relies on the ability of low-grade ultrasounds to detach, but not kill, bacteria adherent to the surface of an implant and, at the end, to permit bacterial culture. Interestingly, sonication seems to be less influenced by a previous antimicrobial therapy than other culture-based diagnostic methods. Amongst other characteristics, sonication is able to detect polymicrobial infections and permits the enumeration of bacteria in the biofilm with, in some circumstances, the possibility of good discrimination between infective and non-infective conditions. Last but not least, SFC is an easy-to-perform and low-cost diagnostic method, which, therefore, may be implemented in all the microbiology laboratories (Table 1).

**TABLE 1:**
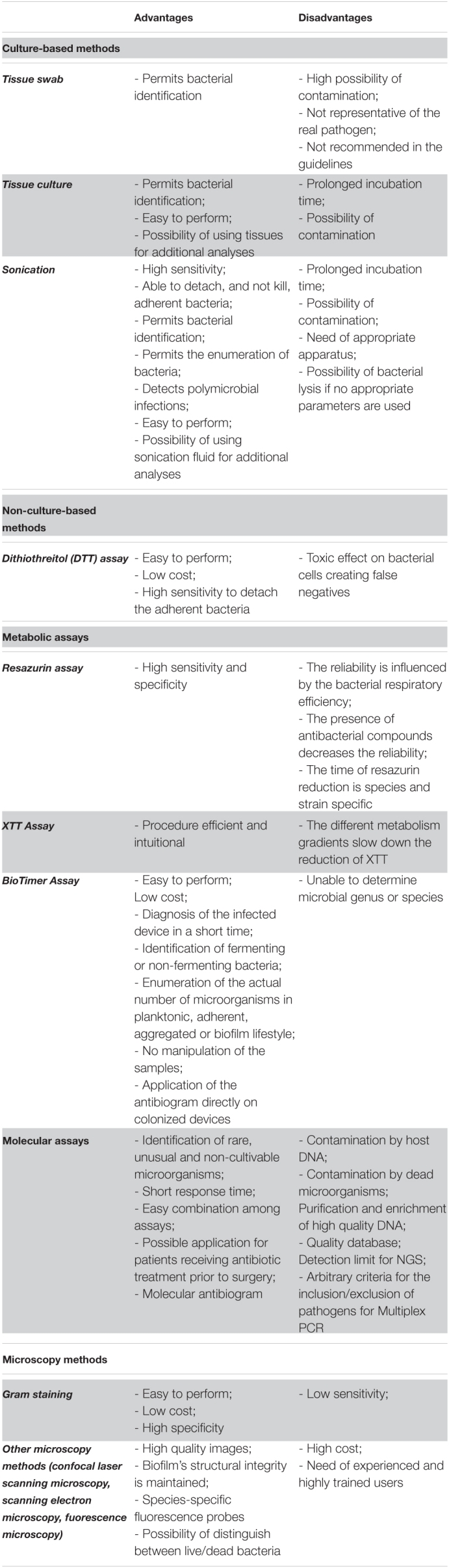
Overview of the principal advantages and disadvantages of culture- and non-culture based methods for the diagnosis of implant-associated infections.

However, it should be highlighted that, as sonication requires multiple processing steps, especially when combined with vortexing and centrifugation, the risk of contamination may occur, frequently caused by low-virulent organisms such as CoNS and *C. acnes*. This also applies if bag leakage during sample collection occurs (Trampuz and Widmer, 2006) and, with this regard, the use of solid and air-tight containers may further reduce the risk of contamination (Trampuz et al., 2007). Therefore, adequate staff training and use of appropriate containers are crucial. Another disadvantage of sonication is represented by the long-lasting incubation period, which may influence the start of adequate antimicrobial treatment. To overcome these limitations, the combination with metabolic or molecular assays may be of high importance in order to shorten the time to pathogen detection.

## Non-Culture Based Methods

### Dithiothreitol Assay

DTT is a strong reducing agent that reduces disulfide bonds at the sulfhydryl group in peptides and proteins. Specifically, by cleaving disulfide bonds between cysteine groups, it acts as a protein denaturant (Olofsson et al., 2003). Based on these considerations, Drago et al. (2012) hypothesized that a new treatment with DTT could be able to remove bacterial biofilm from prosthetic implants. Specifically, in their first pilot *in vitro* study, authors compared the detection rate of DTT compared to that of N-Acetyl cysteine (Drago et al., 2012), scraping and sonication from polyethylene and titanium discs. Treatment with DTT showed a marked increase in bacterial colony counts detection. Detachment of *Pseudomonas aeruginosa* and *Escherichia coli* showed similar yields for DTT and sonication, but lower than for scraping and N-Acetyl cysteine treatment, whereas detachment of *Staphylococcus aureus* and *Staphylococcus epidermidis* was greater for DTT-treated discs than for those treated with sonication, scraping and N-Acetyl cysteine. Overall, these data suggest that the treatment of prostheses with DTT could be useful for the diagnosis of PJIs (Olofsson et al., 2003). To this end, the same authors conducted a study on joint prostheses and compared DTT treatment with sonication and periprosthetic TC. In terms of sensitivity and specificity, DTT provided values of 85.7% and 94.1%, respectively, which were very close to those for sonication (71.4% and 94.1%, respectively), thus representing a valid alternative to sonication in the microbiological diagnosis of PJI (Drago et al., 2013).

Sambri et al. (2018) proposed DTT treatment as an alternative to sonication in a randomized trial that enrolled 232 patients undergoing knee and hip replacements. The aim was to compare DTT treatment and sonication technique with standard TC for the diagnosis of PJIs. As a matter of fact, sonication fluid culture and DTT showed higher sensitivity (89% and 91%, respectively) than TC (79%). Most important, in the group of patients in whom infection was not suspected before surgical intervention, the sensitivity of DTT showed a higher value (100%) than sonication and TC (70% and 50%, respectively). In contrast, no increase in sensitivity was observed among the 3 techniques for cases in which infection was suspected (Sambri et al., 2018). In contrast to Sambri et al. (2018) and Randau et al. (2020) recently reported that DTT fluid cultures were less sensitive than SFC (65% vs. 75%).

Based on the work done by Drago et al., also De Vecchi et al. (2016) conducted a study on periprosthetic tissue samples treated with DTT for the diagnosis of PJIs compared with simple washing in normal saline. Treatment with DTT showed a sensitivity of 88% and specificity of 97.8%, significantly higher than those obtained for saline (72% and 91.1%, respectively) (Drago and De Vecchi, 2017). The same research group has expanded the DTT treatment by enriching it with specific culture broths for aerobic and anaerobic bacteria suggesting that this approach may be useful to increase the detachment of bacteria from biofilm and optimize bacterial growth and PJIs diagnosis (De Vecchi et al., 2017).

More recently, it was shown that, compared to the conventional culture of periprosthetic tissue samples, a commercial device using DTT, the MicroDTTect system, was able to improve the microbiological diagnosis of low-grade PJIs throughout the identification of additional bacteria. Furthermore, it reduced the time to positivity of cultures, especially in the case of *C. acnes* infection (Kolenda et al., 2021).

The advantages of DTT derive from its simplicity of use, i.e., the lack of special instrumentation, the low costs and the possibility to treat both tissues and devices and therefore it may provide valuable additional support to conventional techniques that are used in the diagnosis of IAIs. However, a disadvantage of DTT is represented by the toxic effect on bacterial cells, possibly misreporting the results of the DTT fluid culture and, thus, creating false negatives (Table 1). Additional studies evaluating the role of DTT in IAIs other than PJIs are warranted.

### Metabolic Assays

The identification and enumeration of the actual number of bacteria in biofilms has been a challenge for microbiologists due to lack of exploratory methods (Pantanella et al., 2008). In the last decades, three metabolic assays have been discovered and implemented for the diagnosis of IAIs, including the Resazurin Assay (RA), the 2,3-bis (2-methoxy-4-nitro-5-sulfophenyl)-5-[(phenylamino) carbonyl]- 2H-tetrazolium hydroxide (XTT) assay and the BioTimer Assay (BTA). Herein, the pros and cons of these methods are reviewed (Table 1).

#### Resazurin Assay

The resazurin assay, also named Alamar Blue assay is a simple, rapid, and sensitive measurement for the viability of bacteria. Living cells, metabolically active, are able to reduce, in an irreversible process, the blue-non-fluorescent resazurin (7-hydroxy-3H-phenoxazin-3-one-10-oxide) to the pink-fluorescent resorufin up to a completely reduced colorless state (Pantanella et al., 2013). Pink-fluorescent resorufin can be measured through spectrophotometer. For this purpose, resazurin has been used to determine the actual number of viable cells in biofilm and to detect viable microorganisms in many studies on antimicrobial compounds (Guerin et al., 2001; Peeters et al., 2008; Mariscal et al., 2009).

Recently, the resazurin assay was used to detect 92 colistin-resistant and colistin-susceptible *Acinetobacter baumannii* and *Pseudomonas aeruginosa* isolates. Sensitivity and specificity were 100 and 95%, respectively, compared with the standard broth microdilution method (Lescat et al., 2019). In addition, this assay was used to develop a microplate assay for the evaluation of the antimicrobial activity of electrospun nano fiber filtration membranes for water treatment technologies against the Gram-negative microorganism *Escherichia coli* and the Gram-positive *Enterococcus faecalis* (Travnickova et al., 2019). Resazurin was used as an indicator of the amount of viable microorganisms. Antimicrobial activities of the membranes were evaluated by either resazurin assay or modified ISO 20743 plate count assay. The comparison between resazurin microplate assay and modified ISO 20743 plate count assay showed comparable results, thus indicating that resazurin microplate assay is efficient, faster and less demanding respect to the traditionally one (Travnickova et al., 2019).

However, some limitations have been reported. In fact, the reliability of this assay is influenced by the bacterial respiratory efficiency that, in turn, is conditioned by the microbial growth phase, the age and thickness of the biofilm. Moreover, as the time of resazurin reduction is species and strain-related and some experimental conditions must be standardized. In addition, the presence of antibacterial compounds decreases the resazurin reduction, thus diminishing the reliability of this method in anti-biofilm researches (O’Brien et al., 2000; Mariscal et al., 2009; Sandberg et al., 2009; Skogman et al., 2012; Pantanella et al., 2013).

#### 2,3-Bis (2-Methoxy-4-Nitro-5-Sulfophenyl)-5- [(Phenylamino) Carbonyl]- 2H-Tetrazolium Hydroxide (XTT) Assay

The 2,3-bis (2-methoxy-4-nitro-5-sulfophenyl)-5-[(phenyl amino) carbonyl]- 2H-tetrazolium hydroxide (XTT) is a kind of tetrazolium salt and it is a substrate of mitochondria dehydrogenase. The XTT assay measured the reduction of water-soluble formazan in viable cells. This method uses a redox indicator to enumerate viable cells in biofilm through spectrophotometry (Pantanella et al., 2013). The number of viable bacteria in biofilm is measured through the absorbance of supernatant after the metabolic reduction of XTT. The results allow direct reading of the absorbance measurement, which makes the procedure efficient and intuitional (Adam et al., 2002; Xu et al., 2016). However, the different metabolism gradients present in the heterogeneity composition of biofilm as well as the formation of mature biofilm slow down the reduction of XTT or partially retain it, and, therefore, represent the main limitations of this method (Honraet et al., 2005; Pantanella et al., 2013).

#### BioTimer Assay

**FIGURE 2:**
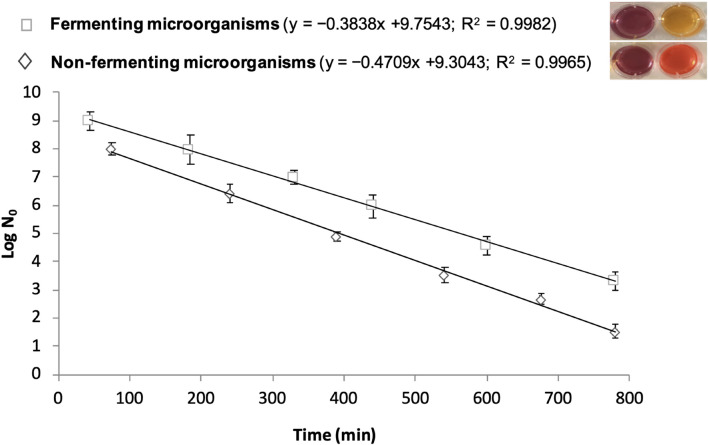
Correlation lines obtained by BioTimer Assay with the reagent containing both Phenol Red and Resazurin as indicators in order to enumerate fermenting (color switch from violet-to-yellow) and non-fermenting (color switch from violet-to-orange) microorganisms. The correlation lines show the relationship between the time (*X*-axis) required for color switch and the initial number of microorganisms (*Y*-axis). The equations and the linear correlation coefficients describing the correlation lines for both fermenting and non-fermenting microorganisms are reported in parenthesis.

BioTimer Assay (BTA) is a metabolic method able to determine the actual number of microorganisms in planktonic, aggregated, adherent and biofilm lifestyle using an original reactive containing Phenol Red or Resazurin as indicators. The Phenol Red changes color from red to yellow indicating the presence of fermenting microorganisms, while Resazurin switches from violet to pink detecting non-fermenting ones (Pantanella et al., 2013; Rosa et al., 2017). The time required for indicators’ switching is related to the initial number of microorganisms (N0) through a genus-specific correlation line described by the following equation: *t* = log (1 + a/N0)/k where “k” indicates the growth rate and “a” is a function of the metabolic product responsible for the indicator switching (Berlutti et al., 2003; Figure 2).

Noteworthy, BTA does not require sample manipulation. As a matter of fact, BTA is a low cost, easy to perform method and has been applied: (i) to quantitatively evaluate bacteria adherent to polyelectrolyte HEMA-based hydrogels (Berlutti et al., 2003); (ii) to evaluate antibiotic susceptibility of biofilm (Pantanella et al., 2008), and (iii) to verify microbiological quality of foods (Giusti et al., 2011).

Recently, BTA has been applied to enumerate adherent bacteria to different medical devices (Rosa et al., 2017, 2019). In particular, BTA was used to evaluate Central Venous Catheters colonization in comparison with the vortex method. 125 Central Venous Catheters removed from patients for suspected Catheter-related Bloodstream Infection or at hospital discharge were examined. BTA was reliable, in 100% agreement with vortex method, in assessing the sterility and catheters colonization and in discriminating fermenting from non-fermenting bacteria (97.1% agreement with vortex method) (Rosa et al., 2017). BTA also shortened the analytical time by 2/3-fold compared with standard culture methods (Rosa et al., 2017). Remarkably, the ascertained Catheter-related Bloodstream Infection diagnosis caused the switch of BTA reagent(s) within 8 h in 18 Central Venous Catheters analyzed (100% agreement) and within 12 h in other 11 catheters analyzed (67% of agreement) (Rosa et al., 2017). In this respect, as blood culture requires more time to confirm Catheter-related Bloodstream Infection due to microbial growth, a rapid change of BTA reagents indicates a high number of colonizing bacteria on Central Venous Catheters thus alerting the physician to the possibility of Catheter-related Bloodstream Infection.

However, BTA is unable to determine microbial genus or species. To overcome this intrinsic limit, the use of BTA, in combination with Vortex-Sonication-Vortex Method for the diagnosis of IAIs has been applied (Rosa et al., 2019). The implants analyzed included CIEDs, Peripherally Inserted Central Catheters, port-a-caths, Central Venous Catheters, and ureteral stents. In addition, a new version of BTA, containing both Phenol Red and Resazurin as indicators, was set-up in order to enumerate both fermenting and non-fermenting microorganisms. If fact, this new reagent is able to selectively distinguish fermenting from non-fermenting microorganisms through the indicators’ color switch from violet-to-yellow and from violet-to-orange, respectively (Rosa et al., 2019). Over 2016–2018, 46 patients with IAIs were enrolled and their 82 explanted devices were analyzed with Vortex-Sonication-Vortex and BTA. In particular, in order to permit the simultaneous analysis by Vortex-Sonication-Vortex and BTA, each device was covered in the BTA reagent instead of the standard saline solution and, successively, each device was vortexed, sonicated, and vortexed again as previously described (Oliva et al., 2016). After Vortex-Sonication-Vortex treatment, a small amount of the suspension was used for classical microbiological analysis and, contemporary, each device immerged in BTA reagent was incubated at 37°C and monitored for the color switch (Rosa et al., 2019). Vortex-Sonication-Vortex plus BTA found microorganisms in 39/46 patients (84.7%) compared with 32/46 (69.5%) and 31/46 (67.3%) by Vortex-Sonication-Vortex and BTA alone, respectively. The combined methods led to microorganism detection in 54/82 devices (65.9%) compared with 43/82 (52.4%) for Vortex-Sonication-Vortex alone and 44/82 (53.6%) for BTA alone (Rosa et al., 2019).

Overall, the combination of both methods permits (i) to diagnose an infected device in a short time; (ii) to identify as fermenting or non-fermenting bacteria and enumerate the actual number of microorganisms directly on the implants without any manipulation of the sample; and (iii) to eliminate false-negative results, thus representing a simple and accurate approach for the identification and enumeration of microorganisms adherent to devices.

Moreover, BTA can be also applied to carry out antibiogram directly on colonized devices without any manipulation (Pantanella et al., 2013).

### Molecular Assays

The culture-based methods are fully effective in the identification and characterization of planktonic bacteria causing systemic infections, whereas they show limitations for identifying bacteria in the biofilm. Currently, most of the microbiological fields, with the exception of clinical microbiology, have shifted from culture to molecular methods. These techniques, which can be performed on the majority of medical devices, can allow the identification of microorganisms even in the case of negative-cultures, occurring for either the presence of non-cultivable microorganisms or a prior antibiotic therapy causing the inhibition of microbial growth. Currently, standard molecular approaches include the extraction of microbial DNA, or RNA, from the specimens, the subsequent amplification by universal or specific Polymerase Chain Reaction (PCR), and sequencing. Among critical steps in such procedures, the employment of efficient lysis buffer to ensure the lysis of all bacterial cells as well as the accurate rinse of the medical device before the extraction of bacterial DNA are imperative to ensure that all and only biofilm bacteria are identified. As a matter of fact, a critical limitation of molecular methods relies on the possible contamination of the specimen by host DNA. On the other hand, also bacterial DNA can contaminate sterile surgical grade irrigation fluids and sampling containers (Burmølle et al., 2010; Swearingen et al., 2016), thus rendering difficult to distinguish between bacterial contamination, which can derive from different sources such as the operating room environment or the patient skin, and actual infective clinical bacteria. In addition, free DNA from dead bacteria can represent a source of contamination, which can be avoided by the use of reverse transcriptase for mRNA amplification, a technique specifically identifying live and active bacteria (Stoodley et al., 2011). To overcome such contamination issue, clinical devices are usually rinsed with large volumes of PBS to remove debris of host tissue as well as planktonic or scarcely adhered bacteria. Indeed, to improve the detection of bacterial DNA in clinical samples, it is pivotal to purify high quality DNA enriched in bacterial source, as a high background of host DNA may hinder the downstream detection of bacterial DNA. Moreover, sequencing process can result in the identification of hundreds of bacterial species in varying amounts, thus requiring a somewhat arbitrary cutoff points to discard false positives. So far, a novel approach for the diagnosis of infections associated to medical devices combines two complementary methods, the sonication of removal implants and subsequent PCR of the resulting sonication fluid (Achermann et al., 2010). Generally, two different amplification-based approaches have been applied to IAIs: (i) broad-range 16S rRNA gene PCR screening followed by sequencing, which allows the identification of any bacterial DNA present in a clinical sample; (ii) multiplex PCR, which targets common causative microorganisms.

#### Broad-Range 16S Ribosomal RNA Gene Polymerase Chain Reaction and Sequencing

PCR is routinely applied in clinical practice, from genetic testing to the identification of infectious agents (Hamady and Knight, 2009; Moure et al., 2011). In microbiological diagnosis, the most targeted gene is the 16S ribosomal RNA (rRNA), as it includes both highly conserved and hypervariable regions: the first serving as target sites for universal bacteria primers whereas the latter used for the identification of bacterial taxa. Moreover, broad-range 16S rRNA amplification has been reported to be a potential tool for the identification of novel microorganisms (Relman et al., 1992; Drancourt et al., 2004; Meddeb et al., 2016), and the combination with sequencing techniques allows the identification of bacterial strains not identified by conventional phenotypic methods or mass spectrometry (Clarridge, 2004; Petti et al., 2005). Similar to standard or quantitative PCR (qPCR), the broad-range 16S rDNA PCR is able to detect both viable and non-viable bacteria, thus representing a useful tool when microbiological techniques give negative results (Rothman et al., 2010; Meddeb et al., 2016). Indeed, the identified bacteria are often rare, unusual, difficult to culture, or bacteria for which a specific PCR is not available (Rothman et al., 2010). However, the breadth of broad-range 16S rDNA PCR makes it susceptible to contamination. In this regard, the amplification of environmental or PCR mixture contaminants (Corless et al., 2000; Aslanzadeh, 2004; Chang et al., 2011) as well as of eukaryotic DNA from the host (Horz et al., 2008; Handschur et al., 2009) represent a criticism for the sensitivity and specificity of such technique. The incidence of false positive can be limited by performing PCR on independent samples from each patient and/or by employing specific primers for pathogen virulence factor genes beside the 16S rRNA gene primers, increasing specificity. Another pivotal drawback regards the pathogen identification which can result circuitous: the use of universal primers for the amplification of 16S rRNA genes will generate a pool of PCR amplicons of all the bacteria present in the sample, thus requiring the sequencing and comparing to known sequences, a lengthy and costly process that requires quality databases (Tzeng et al., 2015). On the other hand, when taxon-specific 16S rRNA gene primers are employed, bacteria not belonging to the plotted taxa will not be detected. Notably, improvements have been developed for both approaches, and wide-ranging databases of human-associated microbes have become available (Human Microbiome Project Consortium, 2012).

The next-generation sequencing (NGS) technique, and in particular the pyrosequencing, enables the parallel and fast identification of bacteria at a much lower cost than traditional Sanger sequencing.

Interestingly, although NGS technique has been widely used to study human microbiome as well as 16S rRNA amplicons to profile the bacterial diversity in a specimen, few studies have employed such methods for the diagnosis of implant-associated infections, including prosthesis joint infection (PJI) and urinary tract catheter-associated infection (Gomez et al., 2012; Xu et al., 2012; Kotaskova et al., 2019). Indeed, conversely to body sites with abundant bacteria, applying NGS to a normally sterile site (such as for medical devices) for the detection of pathogens, can result in significant issues about specimen contamination. Specimens with a high amount of bacteria present less contamination issues than samples with a lower amount since the target will outcompete contaminants in the initial steps of PCR amplification. Moreover, when working with periprosthetic tissue specimens, another crucial issue is represented by the detection limit of NGS, strongly dependent on the DNA extraction efficiency which, in turn, is influenced by the ratio of host to bacterial DNA (Ryu et al., 2014). In these specimens, the recovered amount of bacterial DNA can be very low, due to either the relative abundance of human DNA or inefficient DNA extraction methods, thus globally leading to false negative results.

Hence, in most cases, culture-based methods may represent a better tool for the diagnosis of IAIs than NGS, concerning detection limit, cost, and handling time. However, DNA amplification-based methods can be valuable in cases where culture results negative despite a clinical suspicion of infection, or where rare, difficult-to-culture (i.e., *C. acnes*) or uncultivable bacteria are supposed to be present. Few studies have compared standard and molecular methods for implanted devices (Xu et al., 2012; Ivy et al., 2018; Puges et al., 2018; Thoendel et al., 2018; Esquer Garrigos et al., 2020). In an explorative investigation, Xu et al. (2012) evaluated, by both culture and non-culture based approaches, the bacterial colonization of 55 specimens from patients clinically suspected of having PJIs. NGS analysis and microbiological cultures were concordant for 15/25 specimen sets (60%; five positive, 10 negative), whereas additional taxa were detected by gene analysis in four sets and discrepant data were obtained for six sets (5/6 negative on culture) (Xu et al., 2012). In another study, 408 sonicate fluid samples, from resected knee and hip arthroplasties, were analyzed by metagenomics and compared to results obtained by vortexing/sonication method (Thoendel et al., 2018). The data showed how metagenomics identified known pathogens in 94.8% (109/115) of culture-positive PJIs and new potential pathogens in 43.9% (43/98) of culture-negative PJIs, whereas the detection of microbes in samples from cases of uninfected aseptic failure was rare (3.6%, 7/195 cases) (Thoendel et al., 2018). A similar study, carried out by the same group on 168 failed total knee arthroplasties, reported that genus- and species-level metagenomics detected, respectively, known pathogens in 74 (90%) and 68 (83%) out of 82 culture-positive PJIs, as well as 19 (76%) and 21 (84%) out of 25 culture-negative PJIs (Ivy et al., 2018). The authors conclude that metagenomic shotgun sequencing can be a powerful tool for the identification of a wide range of PJI pathogens, including difficult-to-detect ones in culture-negative infections Taken together, the use of NGS in the clinical diagnosis of infections requires the use of appropriate controls as well as knowledge of the limitations of the chosen method for accurate interpretation of the data. More studies are necessary to define their role in the diagnosis of biofilm-related infections, as well as protocols which describe their use, their application and make their use mainstream in clinical laboratories, in the perspective that they should complement it rather than replace it.

#### Multiplex Polymerase Chain Reaction

Considerable efforts and handling time can be saved by the simultaneous amplification of multiple sequences in a single reaction, a process known as multiplex PCR. Multiplex PCR is based on the combination of different pairs of primer that amplify unique regions of DNA, under a single set of reaction conditions. This requires specific methods for the analysis of each amplification product from the obtained mixture. Such technique is becoming a rapid and convenient tool in both the clinical and the research laboratory. The development of a multiplex PCR requires strategic planning for the optimization of reaction conditions. Multiplex PCRs can present several issues, such as poor specificity, sensitivity or the preferential amplification of specific targets (Zhang et al., 2015; Huang et al., 2018). The use of more than one primer pair makes it prone to spurious amplification products, mainly due to the formation of primer dimers (Brownie et al., 1997). Usually, these unspecific products are more efficiently amplified than the desired target, thus resulting in time- and cost-consuming. In addition, multiplex PCR requires a rational approach for the inclusion/exclusion of specific pathogens in the assay. The choice of the pathogens to be included may depend on the patient’s symptoms or the affected tissue/organ, in relation to the epidemiological characteristics of these pathogens. The pairs of primer must cover as many strains as possible of the target pathogens and should produce amplicons easily to be resolved by using gel electrophoresis or hybridized with maximum specificity (Elnifro et al., 2000). The choice of target species for multiplex PCR assays is dependent on knowledge of the spectrum of bacteria previously linked to PJI, and therefore non-typical bacteria will not be detected by this diagnostic approach. However, this method is expedient and may additionally provide same-day diagnosis, possibly making multiplex PCR diagnostics superior to bacteriological culture. Multiplex PCR has been successfully employed for PJIs in several studies (Renz et al., 2017, 2018; Lausmann et al., 2020), resulting critical for patients receiving antibiotic treatment prior to surgery (Portillo et al., 2012; Cazanave et al., 2013; Ryu et al., 2014; Malandain et al., 2018). Cazanave et al. (2013) found advantageous the combination of broad-range and specific primer pairs for the most common PJI bacteria, like Staphylococci. Morgenstern et al. (2018) reported that, whereas the overall performance of synovial fluid PCR was comparable to culture, multiplex PCR was superior for detection of low-virulent bacteria such as *Cutibacterium* spp. and CoNS. In addition, multiplex PCR provided results within 5 h against several days for synovial fluid culture (Morgenstern et al., 2018). Further, the new generation of multiplex-PCR improves microbial detection, offering the option of faster and targeted antimicrobial therapy, particularly in the context of an acute periprosthetic infection (Lausmann et al., 2020). Interestingly, a recent meta-analysis study demonstrated that PCR of fluid after sonication is reliable and of great value in PJI diagnosis, and that multiplex PCR may improve sensitivity and specificity (Liu et al., 2018).

#### Polymerase Chain Reaction/ESI-MS

A novel alternative to sequencing is represented by electrospray ionization mass spectrometry for the determination of the mass, and therefore base pair compositions and abundances, of PCR amplicons (PCR/ESI-MS). The obtained composition is compared with known bacterial and fungal base compositions to provide the corresponding taxonomic information. Bacteria can then be identified by algorithmic comparison with an extensive database of microbial signature masses (Ecker et al., 2008; Costerton et al., 2011). In addition, a molecular antibiogram can be obtained by including primers for important antibiotic-resistant genes (i.e., mecA for methicillin-resistant *S. aureus*, vanA for vancomycin-resistant Enterococci). The major advantage of using these platforms, of which IBIS T5000 represents the first version, is that all bacterial DNA is amplified by the primer cocktail, rather than just organisms specifically selected, as with cultures and conventional PCR. This makes this technology not only a highly effective diagnostic method, but also an important research tool because new unexpected etiologies will arise from its routine use. So far, this technique has been applied to IAIs in very few studies. Stoodley et al. (2011) were able to identify the presence of *S. aureus*, *S. epidermidis*, and the methicillin-resistant mecA gene in tissue of the removed device from total ankle arthroplasty. Additionally, the Ibis detected that there was close to 10 times more *S. aureus* in comparison to the *S. epidermidis*. Of all the techniques investigated, the authors proposed the IBIS T5000 technology to have the most potential in aiding with clinical detection of PJI with total ankle arthroplasty (Stoodley et al., 2011). Another application was reported for orthopedic surgeons, showing a higher sensitivity than culture-methods in the identification of bacterial pathogens. Of note, it was able to detect bacteria in biofilms when culture was negative, demonstrating its efficacy in case of non-cultivable bacteria from a biofilm (Firoozabadi et al., 2015). Currently, the new version of such technique, the IRIDICA system, has improved the moderate sensitivity showed by IBIS 5000 (around 50–68% vs. culture methods for the identification of bloodstream infections) (Jordana-Lluch et al., 2013), up to 83–91%. Such improvements are due to the optimization of PCR conditions, the increase of blood volume to be tested (5 mL vs. 1.25 mL in the former version) and an ameliorated downstream processing and analysis step to provide high sensitivity (Bacconi et al., 2014).

A correlated technique using matrix-associated laser desorption/ionization time-of-flight (MALDI-TOF) mass spectrometry, in combination with the Biotyper database to detect microbes by their distinct native protein peaks, has been used for the identification of bacterial clinical isolates (Seng et al., 2009; Harris et al., 2010). Further, pyrosequencing and mass spectrometry approaches have been also applied for characterizing microbial antibiotic sensitivity (Seng et al., 2009; Kristiansson et al., 2011).

### Microscopy Methods

#### Gram Staining

Microscopic analysis can be done by means of light microscopy and Gram stain, which may be performed on peri-prosthetic tissues or sonicate fluid. Gram staining is a widely used test for the diagnosis of IAIs, although it is not routinely recommended due to its low sensitivity (Tande and Patel, 2014). Indeed, a meta-analysis conducted by Ouyang et al. (2015) including 18 studies and 4.647 patients found that Gram staining had a very low sensitivity and high specificity (0.19 and 1.00, respectively). Interestingly, the authors suggested that, in the setting of PJIs, Gram stain at revision arthroplasty may guide early antibiotic treatment in case of re-implantation with a preoperative diagnosis of Gram−positive bacterial infection or evidence of purulence (Ouyang et al., 2015). Accordingly, Gram staining alone is not adequate for the microbiological diagnosis of IAIs but it may be used as an adjuvant tool in combination with other diagnostic methods (Ouyang et al., 2015).

Recently, Wouthuyzen-Bakker et al. (2019) evaluated the sensitivity of Gram staining on synovial fluid in late acute *S. aureus* PJIs. Overall, Gram staining was positive for Gram-positive cocci in 59.6% of cases, but the most interesting finding was that Gram staining’s sensitivity was significantly higher when C-reactive protein value at clinical presentation was > 150 mg/L, compared to patients with a lower value (77% vs. 40%, *p* = 0.02), probably due to a higher bacterial inocolum. Accordingly, authors concluded that Gram staining may be a reliable diagnostic tool in late acute PJI to identify *S. aureus* PJI (Wouthuyzen-Bakker et al., 2019).

#### Other Microscopy Methods

Analysis of biofilms on the surface of implants is traditionally performed through culture methods, as described above, or, alternatively, by using crystal violet stain along with spectrophotometry (Wilson et al., 2017). Nevertheless, additional microscopy techniques allowing the visualization of the microbial biofilm may also be considered in the diagnostic approach of IAIs (Høiby et al., 2015). Indeed, methods such as confocal laser scanning microscopy and scanning electron microscopy appear to be very appropriate in revealing biofilms, since they provide images while maintaining biofilm’s structural integrity (Høiby et al., 2015; Grossman et al., 2021). Furthermore, such microscope methods are able to distinguish between planktonic and biofilm bacteria and the identification of the biofilm microorganisms in samples may be obtained throughout species-specific fluorescence *in situ* hybridization probes and fluorescence microscopy (Høiby et al., 2015). With this regard, the application of Peptide Nucleic Acid Fluorescence *in situ* Hybridization on urinary catheter using a universal bacterial and a specific for *Enterobacteriaceae* probes revealed single cells and clusters of *Enterobacteriaceae* within the biofilm, which were further identified as *E. faecalis* and *E. coli* by means of culture methods (Donelli and Vuotto, 2014). Likewise, in animal models, scanning electron microscopy and confocal laser scanning microscopy performed on endotracheal tubes were used for biofilm analysis (Berra and Baccarelli, 2004; Fernández-Barat et al., 2012).

Confocal laser scanning microscopy is a high-resolution technique that allows three-dimensional visualization of biofilm architecture and, when combined with live/dead stain, may serve to quantify biofilm viability; in addition, the presence of extracellular DNA and exopolysaccharides may be visualized (Wilson et al., 2017). Frank et al. (2009) analyzed Foley urinary catheters obtained from patients following total prostatectomy with confocal microscopy and found the presence of dense matrices of microbial cells. Interestingly, microorganisms were most often observed in polymicrobial communities (Frank et al., 2009).

All in all, although the above mentioned microscopy methods possess several characteristics that may render them very useful in the setting of IAIs, they are not routinely available in the laboratory due to their cost and the need of experienced and highly trained users for accurate analyses (Wilson et al., 2017; Table 1).

## Conclusion

With the increasing rate of devices implantation for the treatment of several diseases, a parallel increase in the incidence of IAIs has been observed, leading to an excess of morbidity, mortality, and increased costs for the healthcare system. Dealing with IAIs is a rather complex challenge for treating physicians, and their optimal management requires a multidisciplinary approach and a strict collaboration between different specialists such as surgeons, infectious diseases specialists, microbiologists, pathologists, and radiologists. In particular, a correct microbiological diagnosis of IAIs is of crucial importance in order to prompt an appropriate antimicrobial treatment. Currently, the available diagnostic approaches may be summarized in culture- (i.e., TC and SFC) and non-culture-based methods such as metabolic (DTT, RA, XTT, and BTA) and molecular (Broad-range 16S rRNA gene PCR and sequencing, multiplex PCR and IBIS T5000) ones. Overall, different parameters and conditions may affect the diagnostic yield of each method and, therefore, should be taken into account in the optimization of the diagnostic algorithm of IAIs. These factors include (i) a previous antimicrobial treatment, (ii) the procedure of sample collection (pre-operative vs. intra-operative, number of samples, eventual sample pre-treatment, type of containers), (iii) the used diagnostic method, and (iv) the growth conditions (duration of incubation, type of incubation-aerobic vs. anaerobic-, inoculation into BCBs). As a matter of fact, along with the advantages and disadvantages of each method alone (Table 1), a high diagnostic sensitivity may be obtained by combining these methods with each other, similarly to that already demonstrated (Drago et al., 2013).

The concrete indications for the appropriate use of the described different diagnostic methods may be therefore summarized as follows: (i) in the case of suspected/certain IAI, superficial or tissue swabs must be avoided for their low sensitivity and the risk of contamination, (ii) tissue cultures should be performed, (iii) in the case of implant removal, sonication and quantitative cultures should be applied, and (iv) molecular and metabolic assays should be considered as complementary to culture-based methods to shorten the diagnosis, to search for fastidious microorganisms or when antibiotics have been previously administered.

The research in this field is continuously evolving and, accordingly, a further improvement in the etiological diagnosis of IAIs is expected in the near future.

## Author Contributions

AO, LR, and AC: conceptualization of the narrative review. AO, CMM, DA, FD, MD, LR, and AC: drafting of the manuscript. MV, MTM, PV, and CMM: critical revision of the manuscript for important intellectual content. All authors contributed to the article and approved the submitted version.

## Conflict of Interest

The authors declare that the research was conducted in the absence of any commercial or financial relationships that could be construed as a potential conflict of interest.

## Publisher’s Note

All claims expressed in this article are solely those of the authors and do not necessarily represent those of their affiliated organizations, or those of the publisher, the editors and the reviewers. Any product that may be evaluated in this article, or claim that may be made by its manufacturer, is not guaranteed or endorsed by the publisher.

## Abbreviations

IAIs: Implant-associated infections
PJIs: Prosthetic Joint Infections
CIED-Is: Cardiovascular Implantable Electronic Device Infections
NS-Is: Neurosurgical Infections
US-Is: Ureteral Stent Infections
VG-Is: Vascular Graft Infections
BI-Is: Breast Implant Infections
TC: Tissue Culture
CoNS: Coagulase Negative Staphylococci
SFC: Sonication Fluid Culture
CFU: Colony-Forming Units
EBJIS: European Bone and Joint Infection Society
ICM: International Consensus Meeting
BCB: Blood Cultures Bottles
CSF: cerebrospinal fluid
EVDs: external ventricular drains
VP: ventriculo-peritoneal
MUSC: microbial colonization of the ureteral stent
DTT: Dithiothreitol
XTT: 2,3-bis (2-methoxy-4-nitro-5-sulfophenyl)-5-[(phenylamino) carbonyl]- 2H-tetrazolium hydroxide
BTA: BioTimer Assay (BTA)
PCR: Polymerase Chain Reaction
qPCR: quantitative Polymerase Chain Reaction
NGS: next-generation sequencing (NGS)
OTUs: Operational taxonomic units
MALDI-TOF: matrix-associated laser desorption/ionization time-of-flight.
